# Microplastic Pollution in Surface Water of Urban Lakes in Changsha, China

**DOI:** 10.3390/ijerph16091650

**Published:** 2019-05-12

**Authors:** Lingshi Yin, Changbo Jiang, Xiaofeng Wen, Chunyan Du, Wei Zhong, Zhiqiao Feng, Yuannan Long, Yuan Ma

**Affiliations:** 1 School of Hydraulic Engineering, Changsha University of Science &Technology, Changsha 410114, China; yin@csust.edu.cn (L.Y.); cydu@csust.edu.cn (C.D.); 1576541064@stu.csust.edu.cn (W.Z.); crazy@stu.csust.edu.cn (Z.F.); lynzhb@csust.edu.cn (Y.L.); mayuan1026@gmail.com (Y.M.); 2 Key Laboratory of Dongting Lake Aquatic Eco-Environmental Control and Restoration of Hunan Province, Changsha 410114, China; 3 College of Environmental Science and Engineering, Hunan University and Key Laboratory of Environmental Biology and Pollution Control (Hunan University), Ministry of Education, Changsha 410082, China; wenxf0105@hnu.edu.cn

**Keywords:** microplastic, Changsha, urban lakes, surface water, pollution

## Abstract

As emerging pollutants, microplastics have attracted the attention of scholars from all over the world. However, there is a lack of research on freshwater areas, even in densely populated urban areas. This study investigated eight urban lakes in Changsha, China. It was found that microplastic concentrations ranged from 2425 ± 247.5 items/m^3^ to 7050 ± 1060.66 items/m^3^ in the surface water of research areas and the maximum concentration was found in Yuejin Lake, a tourist spot in the center of the city. Anthropogenic factors are an important reason for microplastic abundance in urban lakes. The major shape of microplastics was linear and most of the microplastics were transparent. More than 89.5% of the microplastics had a size of less than 2 mm. Polypropylene was the dominant type in the studied waters. This study can provide a valuable reference for a better understanding of microplastic pollution in urban areas of China.

## 1. Introduction

Plastic has changed people’s mode of life with its durability and low price. Plastics have grown enormously since the 1950s. Plastic production reached 335 million metric tons in 2016 [1]. According to published research, about 4.8 to 12.7 million tons of plastic garbage is poured into the ocean each year [2]. Plastic has brought convenience into our lives as well as serious environmental problems [3]. Small-sized plastic particles with a diameter of less than 5 mm are defined as microplastics, including primary microplastics with dimensions of less than 5 mm and broken products known as secondary microplastics [4].

Microplastics can be transported to every corner of the world by wind and water currents [5]. Traces of microplastics can be found in remote areas [6,7]. Microplastics also may pose multiple threats to the health of organisms [8]. These particles could scratch animals’ body tissues or fill up digestive organs to form a false sense of fullness, causing them to starve to death. It might also cause stunting or endocrine disorders [9]. All of these microplastics could be accumulated through the food web and ultimately affect human beings [10].

The concern about microplastic pollution in freshwater areas has been booming in recent years [11,12,13,14]. China has the highest plastic production in the world. Microplastic pollution has been reported in lakes, rivers, wastewater treatment plants (WWTPs) and reservoirs in China [15,16,17]. China’s current standard clauses have nothing to do with microplastic pollution and there is a lack of data on the pollution of microplastics in many freshwater areas of China. Changsha is a central Chinese megalopolis with 7.6 million permanent residents. Attention should be paid to the ecological health of the urban aquatic environments in Changsha. Microplastic pollution has been reported in sediments of Changsha [18]. The abundance, distribution and morphological characteristics of microplastics in eight urban lakes of Changsha were investigated in this study. This study can provide a valuable reference for a better understanding of microplastic pollution in freshwater areas.

## 2. Material and Methods

### 2.1. Sample Collection

**Figure 1:**
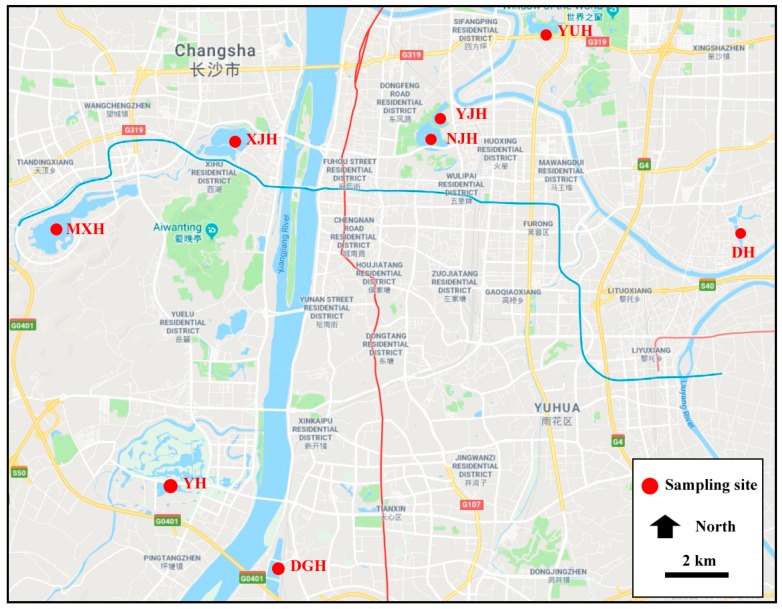
Geographic locations of sampling sites.

There are more than 20 lakes in the urban area of Changsha. This study selected eight major lakes from every district as sampling sites (Figure 1). In each sampling site, 40 liters of surface water sample and three replicates were collected and filtrated with a 45 μm stainless sieve on the sampling spot. Samples were kept with 5% formalin solution at 2 °C before analysis. A series of modified measures were taken in this study. Before analyzing the samples, the workplace was cleaned with 70% alcohol. All apparatuses were rinsed three times with deionized water and covered with tinfoil to avoid airborne lines contamination. A cotton lab coat and nitrile gloves were worn from the beginning to the end of the procedure.

### 2.2. Laboratory Analysis

This study employed an analytical method for the isolation and identification of microplastics which was supported by the National Oceanic and Atmospheric Administration [19]. This method has been used in numerous studies since it was proposed [20,21]. Briefly, samples were treated with 30% H_2_O_2_ to digest natural organic materials, using Fe (II) solution as a catalyst. Each sample was diluted with deionized water and then filtered with a microline filter paper. The filter papers were placed in pre-cleaned petri dishes and inspected visually with a 20–80-fold stereoscopic microscope (SZX7, Olympus, Japan). Microplastic particles were categorized according to their morphological characteristic, including size, shape and color. Microplastics were separated into six size classifications: 50–500 μm, 500–1000 μm, 1000–2000 μm, 2000–3000 μm, 3000–4000 μm and 4000–5000 μm. Microplastics were categorized according to morphology as line, film, foam or fragment. A microplastic was defined as a line if it had a long and thin shape. Thin layer plastic debris was defined as a film. Foam was any kind of polymer material that was dispersed by large numbers of gas microspores in plastics. Plastic debris that did not fit into any of the former three categories was defined as a fragment. The concentration of microplastics was counted as items per cubic meter of water (items/m^3^). In order to further investigate the surface morphology of microplastics collected in the field, surface characteristics of several typical microplastics were investigated with scanning electron microscopy (SEM) (S-4800, Hitachi, Japan) [22,23]. In addition, a subset of 80 particles were selected and verified by micro-Raman spectroscopy (inVia, Renishaw, UK, 532 nm laser, Raman shift 50–3500 cm^−1^).

## 3. Results and Discussion

### 3.1. Abundance and Distribution of Microplastics

Microplastics were found in all surface water samples collected from the urban lakes of Changsha (Figure 1). The concentrations ranged from 2425 ± 247.5 items/m^3^ to 7050 ± 1060.7 items/m^3^ in the water samples (Table 1).

Different concentrations of microplastics can be attributed to a variety of factors, such as hydrological conditions, surroundings around the water and the properties of different plastics [24,25]. The highest concentration of microplastics was found in Yuejin Lake and the second highest concentration of microplastics was found in Nianjia Lake, which is adjacent to Yuejin Lake. Nianjia Lake and Yuejin Lake are located in Martyr Park. Martyr Park is the biggest park in Changsha city and has more than 5 million visitors per year. It is surrounded by dense residential areas and prosperous commercial districts. It is a populous location in the city center. Plastic garbage from tourists and nearby residents is broken by various external effects. The stability properties of plastics cause them stay in the surface water. As the two artificial lakes have no open channel flow, microplastics are more easily suspended in both lakes. Another high concentration of microplastics was found in Donggua Lake, a fishery near the second ring road of Changsha. Many local restaurants around Donggua Lake use fishing nets to pull fish up and attract customers with delicious meals of fish. The breeders kill fish on the spot and take them away in plastic bags. Fragments of fishing nets and the degradation of plastic bags might be the most significant sources of microplastics in Donggua Lake [26]. Wastewater treatment plants (WWTPs) could be a source of the microplastics in nearby lakes. Dong Lake is located near both Huaqiao wastewater treatment plant and Langli wastewater treatment plant, thus leading to a relatively high concentration of microplastics. Lakes located on the west bank of Xiangjiang River, such as Yang Lake, Meixi Lake and Xianjia Lake, had relatively low abundances of microplastics. This phenomenon could be attributed to several reasons. These lakes are located in Yuelu District, which is the western urban area of Changsha. A large part of Yuelu District is mountains, though there are also wetland parks and university campuses with lower population densities. Similar microplastic levels were found in Yue Lake, which is located in the north edge of Changsha. Published research considered that anthropogenic activity is an important reason for microplastic pollution [27,28]. Large amounts of wastewater and litter enter the surface water through various ways. This phenomenon is reflected in published studies, as high concentrations of microplastics were recorded in lakes located in populous areas, such as the Laurentian Great Lakes, Northern America [29] and Bei Lake, Wuhan, China [30]. Relatively remote sites, by contrast, found lower microplastic concentrations, such as Lake Hovsgol, Mongolia [31], and Zurich Lake, Switzerland [32].

### 3.2. Morphological Characteristics

**Figure 2:**
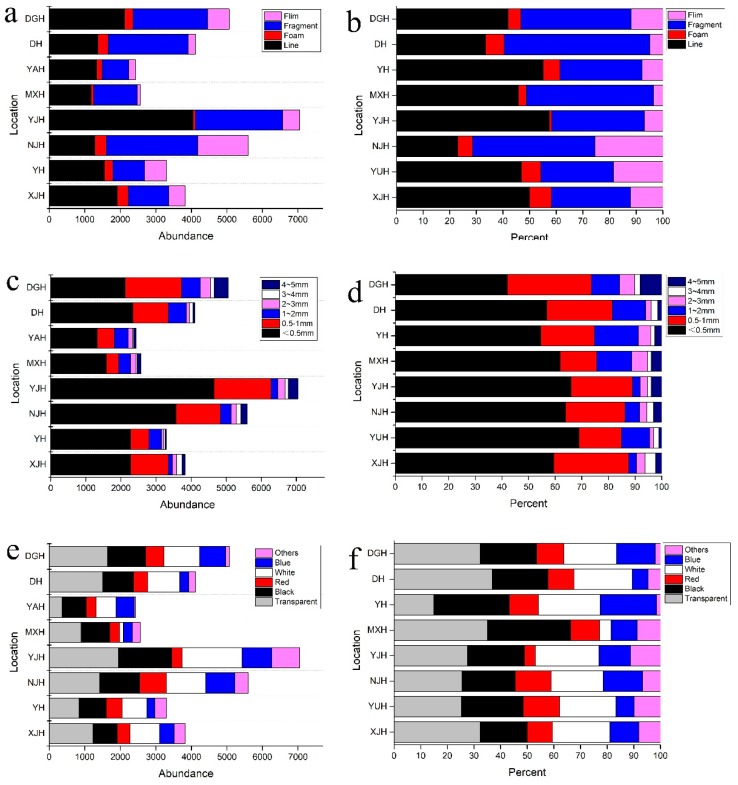
Abundance and proportion of (**a**,**b**) type distribution, (**c**,**d**) size distribution and (**e**,**f**) color distribution of microplastics collected from Changsha.

Four types of microplastics (line, film, foam and fragment) were found in the collected surface waters of Changsha (Figure 2a,b).

**Figure 3:**
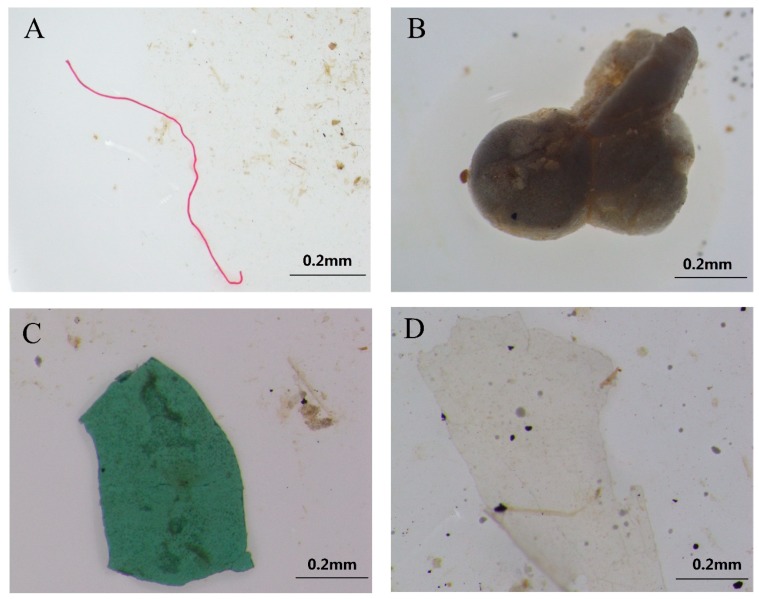
Typical photographs of microplastics: (**a**) line, (**b**) foam, (**c**) fragment, (**d**) film.

Figure 3 presents photographs of four types of typical microplastics. Lines accounted for the largest number of microplastics. Many behaviors in the lives of citizens could cause lines to enter surface water, such as washing clothes and daily cleaning [33,34]. Because WWTPs cannot completely remove lines from domestic sewage, domestic sewage has become another important source of microplastics in surface water. In some lakes used as fisheries, like Donggua Lake, lines from fishing nets and other fishing gear also cannot be ignored [35]. The amount of line microplastics was followed by fragments and films. Like other megalopolises in China, Changsha has more than 7 million permanent citizens relying heavily on express deliveries and take-out food in their daily lives, which results in a huge amount of disposable plastic products from packaging. Most used plastics are not recycled or properly disposed of, causing them to be broken down or cracked into microplastics for various reasons [36]. Then the microplastics enter the surface water in large quantities, retaining their stable properties. In addition, living standards are high in this developed city, and thus plastic is an important material for furniture, containers, toys, cosmetics and other daily necessities. This represents another possible source of microplastics. Hard plastic and outer packaging might be the source of fragments. Plastic bags might be the main source of films [4,37]. Although the amount of foam was much lower than the amounts of lines, fragments and films, it was still detected in all lakes. Foam has a special structure with many voids inside and it is often used for sound insulation, heat insulation and collision avoidance. It is widely used in packaging, automobile components, clothing and building implements. The fragile and low density properties make it easier for foam to enter and float in surface water [37]. In terms of size, 50–500 μm accounted for the highest proportion in all sampling sites (Figure 2c,d). Results showed that there was a higher proportion of smaller-size microplastics. This might be attributed to the fact that a large piece of plastic could be broken into multiple smaller-sized plastics. Microplastics smaller than 2000 μm accounted for 89.5%, which was similar to the findings of published studies on freshwater areas such as the Laurentian Great Lakes, USA [29], Wuhan urban lakes, China [30] and Three Gorges Reservoir, China [38]. Another way to categorize microplastics is by their color. Microplastics were categorized according to their color and divided into six color classifications: transparent, black, white, red, blue and other (Figure 2e,f). Transparent particles were the most dominant in all samples. An important source of transparent particles might be plastic bags, used extensively by citizens in their in daily lives. Many colored particles, especially some lines and films, might lose color during or after entering surface water. Black and white particles were also widely distributed in all samples. Compared with transparent microplastics, colored microplastics are more likely to be swallowed by aquatic organisms, which will cause damage to their health [39]. The impact of colored microplastics on aquatic life deserves more attention in the future.

**Figure 4:**
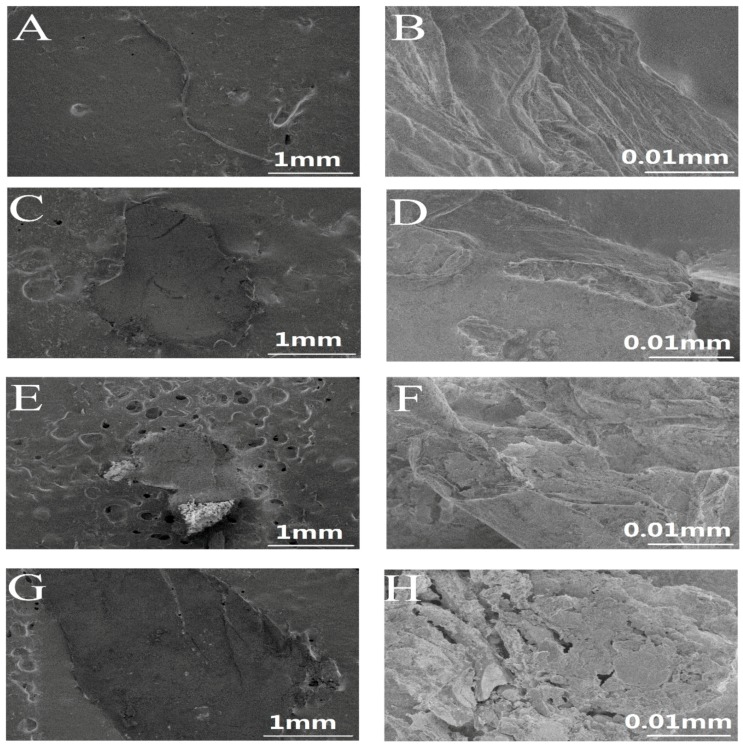
SEM images of microplastics: (**a**,**b**) line, (**c**,**d**) fragment, (**e**,**f**) foam, (**g**,**h**) film.

Four typical microplastics photographed by a scanning electron microscope (SEM) are shown in Figure 4. In outdoor environments, microplastics are affected by weathering or other external forces, often with pits or cracks on the surface as shown in Figure 4. Therefore, the microplastics found in water often have a larger body surface area, which can make them better carriers for microorganisms or other contaminants.

### 3.3. Composition of Microplastics

**Figure 5:**
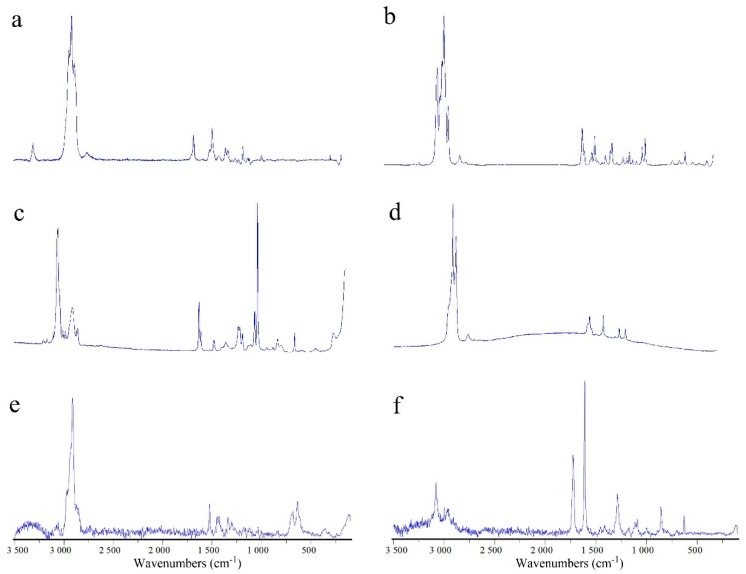
Typical micro-Raman spectra of the randomly selected microplastics. (**a**) PA, (**b**) PP, (**c**) PS, (**d**) PE, (**e**) PVC, (**f**) PET.

A total of 80 particles (randomly selected from each sample site) were identified and their polymers were determined with micro-Raman spectroscopy, which is widely used to identify the polymers of unknown particles [38,40]. The results of the micro-Raman spectroscopy analysis are shown in Table 2. Typical Raman spectra are shown in Figure 5. A total of six kinds of microplastics with different chemical components were found: Polypropylene (PP), polyethylene (PE), polystyrene (PS), polyethylene terephthalate (PET), polyamide (PA) and polyvinyl chloride (PVC).The high cost of micro-Raman spectroscopy identification meant that we did not detect the complete composition of collected particles. This may have caused our results to deviate from the actual situation. It is necessary to find more economical, accurate and rapid identification methods in the future in order to obtain more detailed and accurate experimental results.

Polypropylene was the highest proportion of all types, followed by polyethylene, polystyrene and polyethylene terephthalate. A few particles were identified as polyamide and polyvinyl chloride. There were also two particles that were not plastic; they might have been metal wire or cotton. Polypropylene, with low density, excellent mechanical properties, heat resistance, stable chemical properties and great electrical insulation, has been widely used in household appliances, pipes and packaging. Polyethylene terephthalate can be spun into polyester lines, which is the common raw material for clothes, fishing nets, carpets and beverage bottles [16]. Polyvinyl chloride is used in bulk for building materials, packaging and was used in disposable medical supplies in earlier years. However, non-toxic polyvinyl chloride releases toxic plasticizers under certain conditions, such as dioctyl phthalate and dibutyl phthalate. The production and consumption of polypropylene has increased rapidly in recent years to replace polyethylene terephthalate and polyvinyl chloride. Polyethylene is widely used in clothing, food containers, fishing nets and mulch. Polystyrene is needed in many light industries, especially foamed plastics [40]. All foams identified by micro-Raman spectroscopy in this study were polystyrene. Polyamide is used mainly as a raw material for synthetic lines that can be used in the daily life of citizens, industrial production, medical treatment and in the arms industry. The results of identification were similar in some areas, such as the surface water of Wuhan City, China [30] and the Three Gorges Reservoir, China [38], but we still found fewer kinds of microplastics than has been reported for some other places, such as Taihu Lake, China [41]. 

Different types of plastics have different densities. The densities of polypropylene and polyethylene are lower than that of freshwater, at 0.92 g/m^3^ and 0.95 g/m^3^. These two types of microplastics were the most common in the surface water of Changsha. Moreover, polyethylene terephthalate, polystyrene, polyamide and polyvinyl chloride have higher densities than freshwater at 1.37 g/m^3^, 1.05 g/m^3^, 1.15 g/m^3^ and 1.3 g/m^3^, respectively, which were also detected in surface waters. The vertical distribution of microplastics in water depends not only on density, but also on other factors including various external conditions, such as hydraulic conditions, salinity, temperature and wind [42]. In addition, microplastics themselves change in some situations. Microplastics can be torn or degraded by external forces, resulting in a change of the surface to volume ratio. This makes it easier for the microplastics to stay on the surface water. Furthermore, a higher surface to volume ratio and rips on the surface will make parasites more prone to attach to microplastics, leading to more complex changes [5,43].

## 4. Conclusions

China has the largest population and the highest plastic production in the world. Populous urban areas suffer heavier microplastic pollution than oceans; however, there is a serious lack of research. This study investigated the occurrence, distribution and composition of microplastics in a metropolis in China and found that the urban lakes in Changsha were universally polluted by microplastics. The most common microplastics were lines, transparent and tiny-sized particles. Polypropylene was the dominant polymer type. There are still many gaps and deficiencies that need to be paid more attention. The sampling methods, separation protocols and assessments of microplastic pollution have problems that need to be improved. This study helps to fill the gaps in the study of microplastic pollution in urban areas and provides a basis for further the in-depth study of inland freshwater areas.

## Figures and Tables

**Table 1:** Information about the sampling sites in this study.

Location	Code	Latitude	Longitude	Abundance (items/m^3^)
Xianjia Lake	XJH	112°56′43.96″	28°12′52.44″	3825 ± 388.9
Meixi Lake	MXH	112°54′29.56″	28°11′44.08″	2563 ± 548.0
Yang Lake	YH	112°5′66.45″	28°7′59.15″	2425 ± 247.5
Yue Lake	YUH	113°2′25.01″	28°14′37.96″	3300 ± 424.3
Yuejin Lake	YJH	113°0′45.47″	28°13′2.99″	7050 ± 1060.7
Nianjia Lake	NJH	113°0′34.99″	28°13′11.29″	5600 ± 1555.6
Dong Lake	DH	113°5′53.70″	28°11′48.21″	4113 ± 247.5
Donggua Lake	DGH	112°57′44.56″	28°6′26.71″	5063 ± 1891.5

**Table 2:** Polymer types from the results of the micro-Raman spectroscopy.

Type	Line	Foam	Film	Fragment	Total	Percentage (%)
PET	6	0	3	0	9	11.25
PP	12	0	5	10	27	33.75
PE	9	0	6	7	22	27.5
PA	6	0	0	0	6	7.5
PS	0	5	1	5	11	13.75
PVC	0	0	0	3	3	3.75
Non-plastic	2	0	0	0	2	2.5
Total	35	5	15	25	80	100
